# Bibliometric and visual analysis of single-cell sequencing from 2010 to 2022

**DOI:** 10.3389/fgene.2023.1285599

**Published:** 2024-01-11

**Authors:** Ling Chen, Yantong Wan, Tingting Yang, Qi Zhang, Yuting Zeng, Shuqi Zheng, Zhishan Ling, Yupeng Xiao, Qingyi Wan, Ruili Liu, Chun Yang, Guozhi Huang, Qing Zeng

**Affiliations:** ^1^ Department of Rehabilitation Medicine, Zhujiang Hospital, Southern Medical University, Guangzhou, China; ^2^ Guangdong Provincial Key Laboratory of Proteomics, Department of Pathophysiology, School of BasicMedical Sciences, Southern Medical University, Guangzhou, China; ^3^ School of Rehabilitation Medicine, Southern Medical University, Guangzhou, China; ^4^ Dongguan Key Laboratory of Stem Cell and Regenerative Tissue Engineering, Guangdong Medical University, Dongguan, China

**Keywords:** single-cell sequencing, VOSviewer, CiteSpace, visual analysis, bibliometric

## Abstract

**Background:** Single-cell sequencing (SCS) is a technique used to analyze the genome, transcriptome, epigenome, and other genetic data at the level of a single cell. The procedure is commonly utilized in multiple fields, including neurobiology, immunology, and microbiology, and has emerged as a key focus of life science research. However, a thorough and impartial analysis of the existing state and trends of SCS-related research is lacking. The current study aimed to map the development trends of studies on SCS during the years 2010–2022 through bibliometric software.

**Methods:** Pertinent papers on SCS from 2010 to 2022 were obtained using the Web of Science Core Collection. Research categories, nations/institutions, authors/co-cited authors, journals/co-cited journals, co-cited references, and keywords were analyzed using VOSviewer, the R package “bibliometric”, and CiteSpace.

**Results:** The bibliometric analysis included 9,929 papers published between 2010 and 2022, and showed a consistent increase in the quantity of papers each year. The United States was the source of the highest quantity of articles and citations in this field. The majority of articles were published in the periodical *Nature Communications.* Butler A was the most frequently quoted author on this topic, and his article “*Integrating single-cell transcriptome data across diverse conditions, technologies, and species*” has received numerous citations to date. The literature and keyword analysis showed that studies involving single-cell RNA sequencing (scRNA-seq) were prominent in this discipline during the study period.

**Conclusion:** This study utilized bibliometric techniques to visualize research in SCS-related domains, which facilitated the identification of emerging patterns and future directions in the field. Current hot topics in SCS research include COVID-19, tumor microenvironment, scRNA-seq, and neuroscience. Our results are significant for scholars seeking to identify key issues and generate new research ideas.

## 1 Introduction

Cells are considered the fundamental unit of biological structure and function (Bai et al., 2021). Research in scientific fields, such as reproductive development, genetics, and neural activity, are rooted in the study of cells. Furthermore, understanding the pathogenic mechanisms of all disorders requires exploration of cytopathic conditions. Cell research is not only the foundation of life science but also a crucial factor in the advancement of modern life science. A research project designated the Human Cell Atlas was initiated to acquire high-resolution information on cell type, number, location, relationship, and molecular expression, as well as accurately describe and define cell composition and health status in diseases. The human cell map is based on molecular maps (such as gene expression) to identify all cell types and associate this information with classic cell characteristics, such as location and morphology (Regev et al., 2017). This research has been recognized as a milestone of epoch-making significance in the field. Recent decades have seen significant progress in sequencing technologies. Traditional sequencing methods, such as Sanger and next-generation sequencing (NGS), have certain limitations. The low-throughput capacity and efficiency of Sanger sequencing make it difficult to meet the needs of modern scientific development for the acquisition of biological gene sequences. NGS has compensated for the shortcomings of Sanger sequencing to an extent. However, this technique generates short reads, which are not desirable for full genome assembly. SCS further addresses the limitations of prior techniques. This high-throughput approach facilitates analysis of the genome, transcriptome, epigenome, and other genetic data at the level of a single cell, providing critical insights into cell type, function, status, and variations. Since its inception, SCS has been extensively utilized in multiple disciplines, such as neurobiology and cancer biology. Brady et al. (1990) originally reported the single-cell cDNA amplification method in 1990. Subsequently, the group of Tang et al. (2009) conducted pioneering research on single-cell mRNA sequencing, which garnered substantial attention. Interest in research on SCS has grown significantly over the past few years. Bibliometric analysis of SCS is therefore essential to provide important insights for harnessing the full potential of completed research and identifying emerging trends in the field.

Bibliometric analysis is a valuable tool for quantitative evaluation of scientific publications and characterization of research trends (Niu et al., 2021). Compared to systematic literature review, bibliometrics provides a more objective and reliable analysis (Aria and Cuccurullo, 2017), which reduces the potential bias caused by subjective intention. The results are of great significance in identifying potential hotspots and avenues for future research in specific fields (Niu et al., 2021). To date, limited bibliometric studies have been conducted in the discipline of SCS. With the increasing number of publications in this area, bibliometric techniques provide an effective means to update the collected data and identify trends in research.

Here, we have conducted a comprehensive bibliometric analysis of SCS studies published between 2010 and 2022 using CiteSpace and VOSviewer, with the aim of visually analyzing research trends in SCS up to this time through evaluation of nations/regions, research institutions, authors, and co-cited authors. Furthermore, we have identified key hotspots and speculated on the future of this research avenue.

## 2 Materials and methods

### 2.1 Data sources and search strategy

**FIGURE 1:**
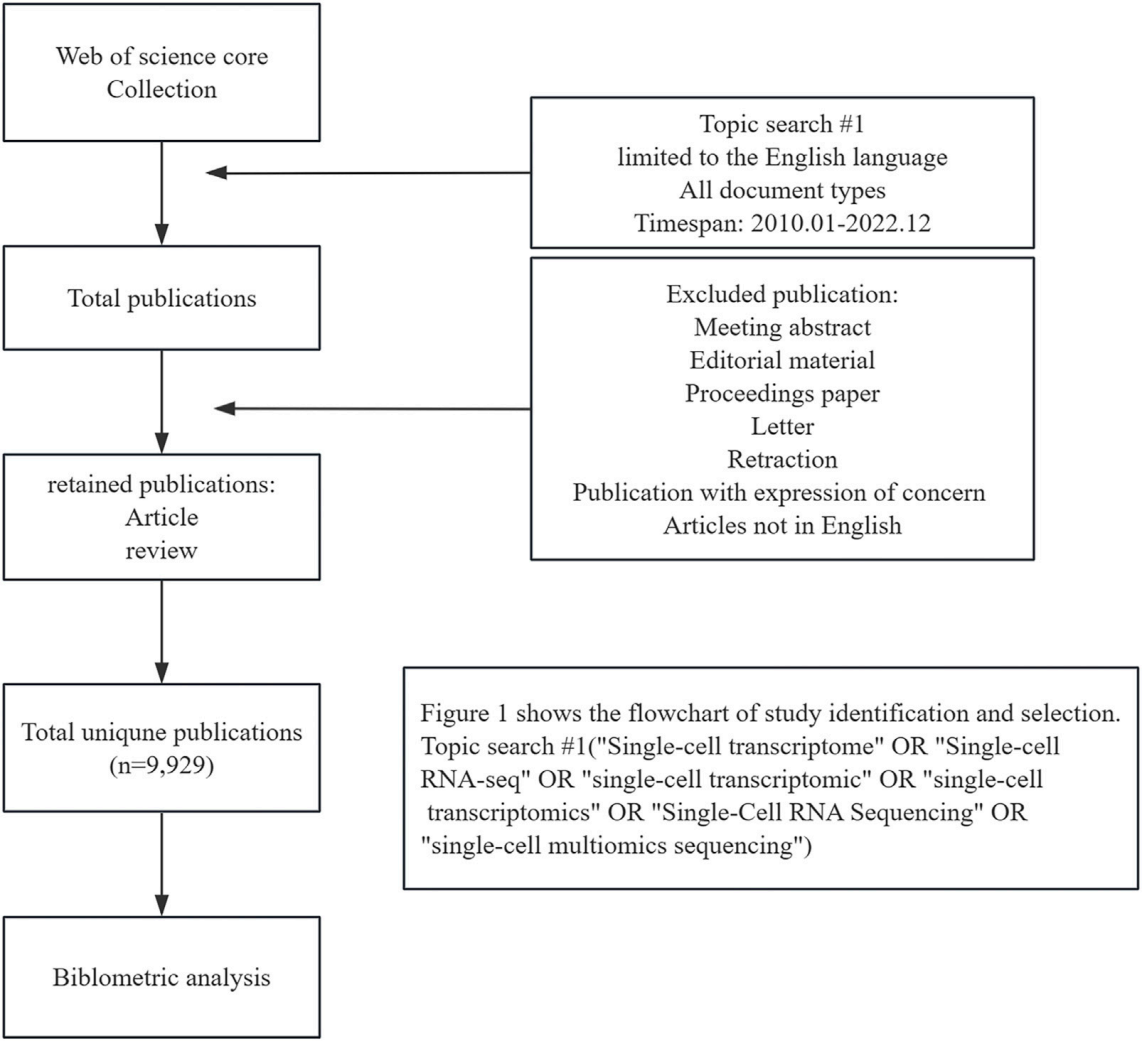
Flow chart of the screening process for research on single-cell sequencing.

Web of Science Core Collection (WOSCC) serves a key global data source for literature searches. We conducted a thorough search of the WOSCC database for research publications between 1 January 2010 and 3 December 2022. On 3 December 2022, we performed a literature search and downloaded data to eliminate potential biases resulting from frequent database updates. The following search strategy was used: TS = (“Single-cell transcriptome” OR “Single-cell RNA-seq” OR “single-cell transcriptomic” OR “single-cell transcriptomics” OR “Single-Cell RNA Sequencing” OR “single-cell multiomics sequencing”). This report focuses on published articles and reviews related to SCS that are limited to the English language. A total of 9,929 records were selected for analysis. The specific literature screening process is presented in Figure 1.

### 2.2 Data analysis

We exported and stored 9,929 documents using Excel and plain text files. To obtain detailed information, relevant data (such as titles, authors, organizations, nations/regions, citations, and periodicals) were collected from the included papers and loaded into VOSviewer, CiteSpace, and the bibliometric analysis software “bibliometrix” for further analyses.

CiteSpace was utilized to conduct network analysis of the potential trends and hotspots, and obtain crucial information on scientific research pertaining to a specific subject (Chen, 2006). CiteSpace version 5.8 was applied in this study to display the progression of research on SCS based on available information, such as authors, institutions, and nations. The Time Slicing specifications for CiteSpace were set to consider each year between 2010.01 and 2022.12 as a timestamp.

VOSviewer (version 1.6.15), a broadly utilized graphical tool that supports various analyses (van Eck and Waltman, 2010), including author co-occurrence, keyword co-occurrence, co-cited literature analysis, and coupling analysis (such as literature, source journals, authors, and institutions), was employed. This information can aid in identifying hotspots in a specific field by highlighting data trends and patterns. The author, institution, nation, and subject attributes were examined via co-occurrence and cluster analyses.

In addition to the above methodologies, quantitative data on the distribution of journals, nations, institutions, authors, and publications on SCS were examined with the aid of “bibliometrix” (https://www.bibliometrix.org), a tool in R (Aria and Cuccurullo, 2017).

## 3 Results

### 3.1 Annual publications and citation trends

**FIGURE 2:**
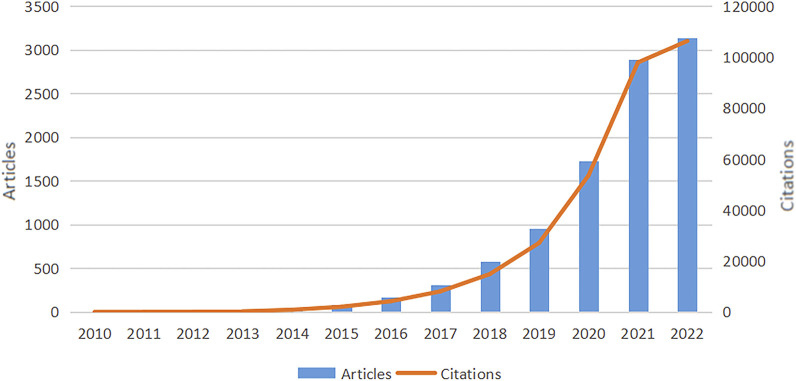
Overall growth in WOSCC articles and citations from 2010 to 2022. The trend of annual publications on research of SCS increased each year between 2010 and 2022, with the publications and citations related to SCS reaching their peak in 2022. The data for 2022 is incomplete.

The quantity of papers and annual citations indicates trends in research directions in this field. We observed an overall increase in the full count of articles and citations in WOSCC between 2010 and 2022, as shown in Figure 2. Prior to 2015, research on SCS was slow to develop, which was followed by a steady rise in annual publications and citations after this time. Although the data are incomplete, the 2022 report recorded the highest frequency of annual citations of 97,860 with 2,890 articles. In conclusion, the field of SCS is evidently a focus of escalating research attention.

### 3.2 Distribution of nations/regions

**FIGURE 3:**
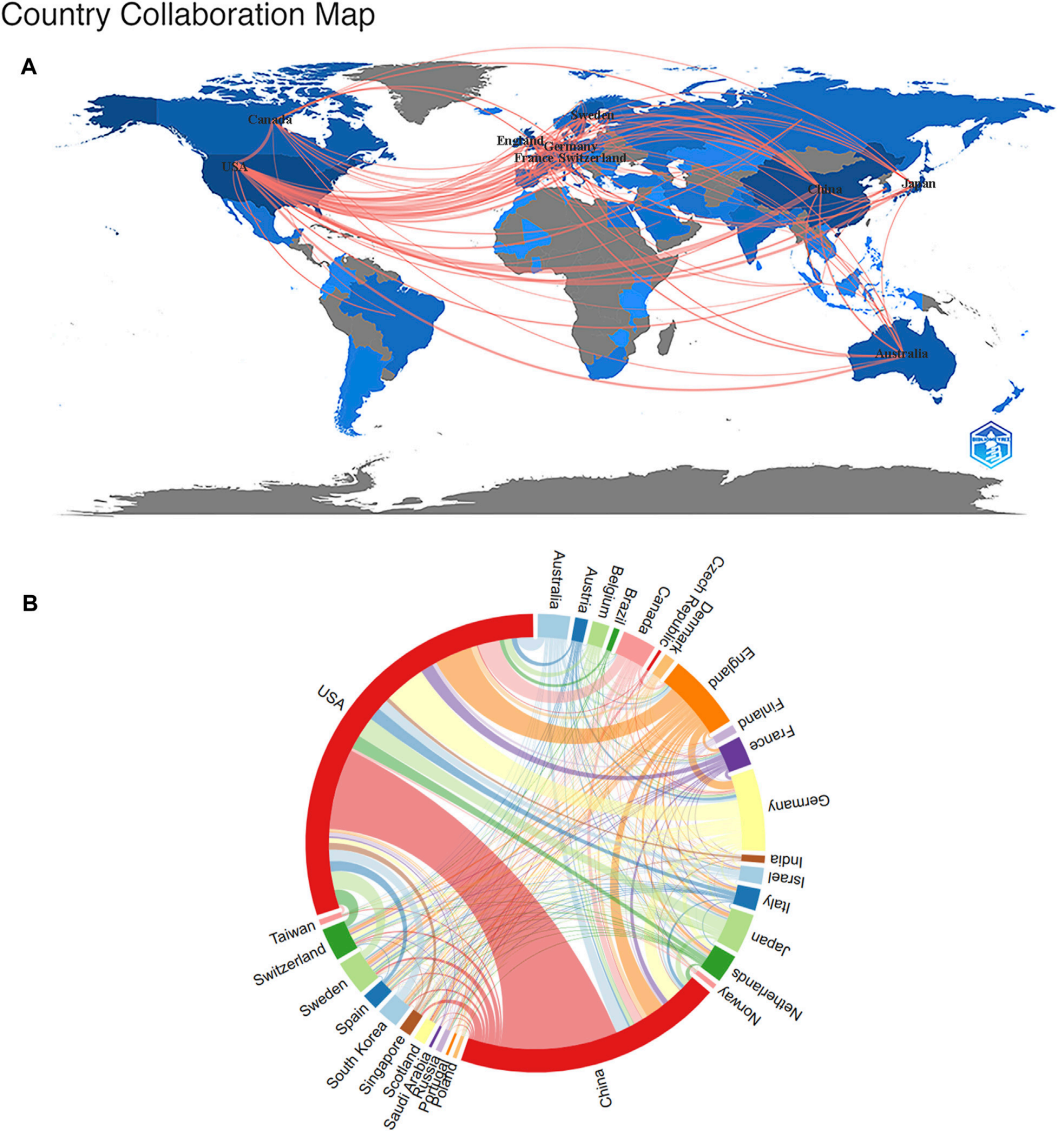
The Analysis of the nations/regions related to SCS. **(A)** Nations/regions collaboration map. Higher rates of collaboration were indicated by darker blue, and the larger the connecting line, the greater the rate of cooperation between nations. **(B)** Distribution of nations/regions related to SCS. The circle diameter indicates the quantity of publications published by each countries/regions, while the lines indicate the level of collaboration among the countries/regions.

Currently, dozens of countries/regions are involved in SCS research. According to Figure 3A, connections were mainly identified between North America and Europe, North America and East Asia, and Europe and Oceania. The top ten nations/regions based on the quantity of articles published, frequency of citations, and link strength are shown in Table 1. The top three countries/regions for SCS publications were the United States, China, and Germany, accounting for approximately 62.72% of all SCS-related articles. The country with the most published papers was the United States (4,919 papers, 49.54%), followed by China (3,120 papers, 31.20%) and Germany (954 papers, 9.61%). The United States remained the most influential country/region in terms of overall citations.

**TABLE 1:** Citation, and link strength rankings by country/region.

Rank	Countries	Publications (%)	Countries	Citations	Countries	Total link strength
1	United States	4,919(49.54%)	United States	197,269	United States	3,300
2	China	3,120(31.42%)	England	48,651	Germany	1,481
3	Germany	954(9.61%)	China	47,852	China	1,446
4	England	884(8.90%)	Germany	43,496	England	1,398
5	Japan	463(4.66%)	Sweden	29,161	Sweden	700
6	Sweden	404(4.07%)	Israel	16,008	Netherlands	617
7	Canada	392(3.95%)	Netherlands	15,962	Switzerland	597
8	Switzerland	382(3.85%)	France	15,390	Canada	573
9	Australia	374(3.77%)	Australia	14,440	France	571
10	France	342(3.44%)	Switzerland	14,315	Australia	556

Cluster analysis is one of the most common methods of multiparametric data analysis, a frequently used procedure for partitioning data into structurally distinct states. Cluster analysis reveals the internal structure of the data, grouping individual observations according to their degree of similarity. A cluster analysis of countries/regions related to SCS was carried out using VOSviewer (Figure 3B). The diameter of the circle indicates the number of publications by country/region while the line signifies the level of cooperation between countries/regions. Our results showed that the United States occupied the largest proportion within the circle, followed by China, Germany, England, Canada, and Switzerland (Figure 3B).

### 3.3 Analyses of institutions

The leading institutions that published the highest quantity of SCS articles are presented in Table 2. The institution with the most articles was identified as Harvard Medical School (512 publications, 5.16%). The other top institutions were the Chinese Academy of Sciences (355 publications, 3.58%), Stanford University (305 publications, 3.07%), and the Karolinska Institute (282 publications, 2.84%).

**TABLE 2:** Top 10 intitutions related to single-cell sequencing.

Rank	Institution	Count (%)	Country	Institution	Total link strength
1	Harvard Med Sch	512(5.16%)	United States	Harvard Med Sch	1,541
2	Chinese Acad Sci	355(3.58%)	China	Broad Inst Mit and Harvard	897
3	Stanford Univ	305(3.07%)	United States	Mit	843
4	Karolinska Inst	282(2.84%)	Sweden	Chinese Acad Sci	667
5	Univ Cambridge	274(2.76%)	United Kingdom	Massachusetts Gen Hosp	635
6	Shanghai Jiao Tong Univ	254(2.56%)	China	Harvard Univ	599
7	Peking Univ	249(2.51%)	China	Brigham and Womens Hosp	585
8	Univ Chinese Acad Sci	240(2.42%)	China	Stanford Univ	555
9	Fudan Univ	233(2.35%)	China	Univ Chinese Acad Sci	525
10	Mit	223(2.25%)	United States	Univ Calif San Francisco	508

**FIGURE 4:**
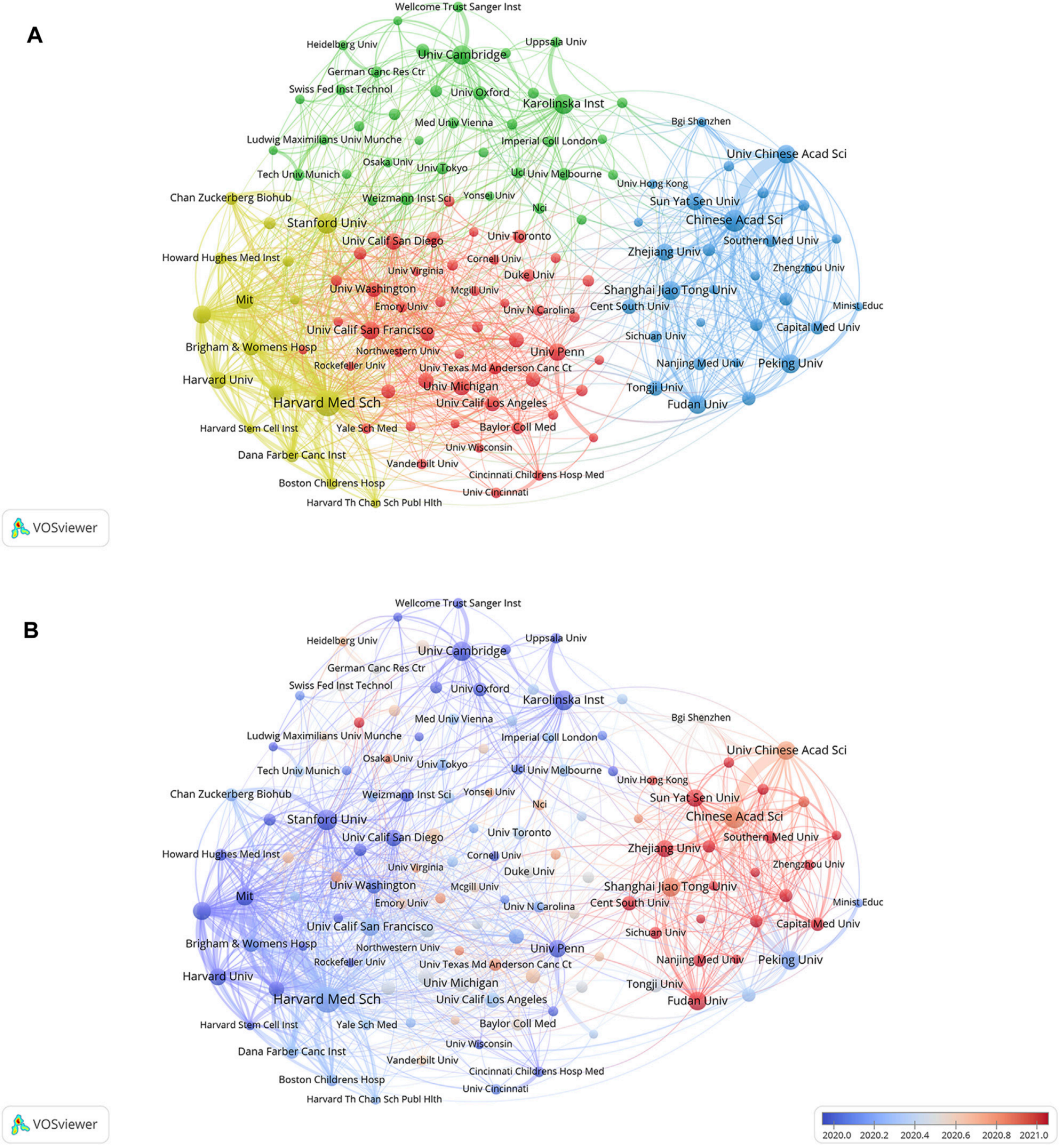
The cluster analysis of academic institutions. **(A)** Analyses of institutions clustering. Node colors denote various clusters, whereas node diameter denotes the quantity of articles produced by institutions and line thickness denotes the degree of institutional collaboration. **(B)** Analyses of the amount of publications institutions have published recently. Blue indicates that the institution has produced fewer papers in recent years, while red indicates that they have produced more.

Cluster analysis of academic institutions was conducted with the aim of comprehending the global distribution of research related to SCS and its connected disciplines (Figure 4A). VOSviewer divides institutional cooperation into four closely related blocks. The node diameter reflects the level of productivity of the institutions while line width indicates the degree of institutional cooperation. The colors of the nodes represent various clusters. It is evident that the institutions are significantly interconnected and engage in frequent communication. Among these institutions, Harvard Medical School, Broad Institute of MIT, and Harvard had the most collaborations, followed by the Chinese Academy of Sciences and Massachusetts General Hospital. VOSviewer was used to generate a heatmap of each institution, as depicted in Figure 4B. Institutions conducting significant research in the discipline of SCS over recent years, and considered an emerging force in the field are presented in red. Institutions that have conducted relatively little research in SCS lately are indicated in blue. Sun Yat-sen University, Zhejiang University, Central South University, Fudan University, and Capital Medical University were identified as the institutions contributing to the majority of research in recent years, followed by Stanford University, Harvard Medical School, Cambridge University, and Karolinska Institute, highlighted as the sources of relatively more research in the past.

### 3.4 Analyses of authors and co-cited authors

**FIGURE 5:**
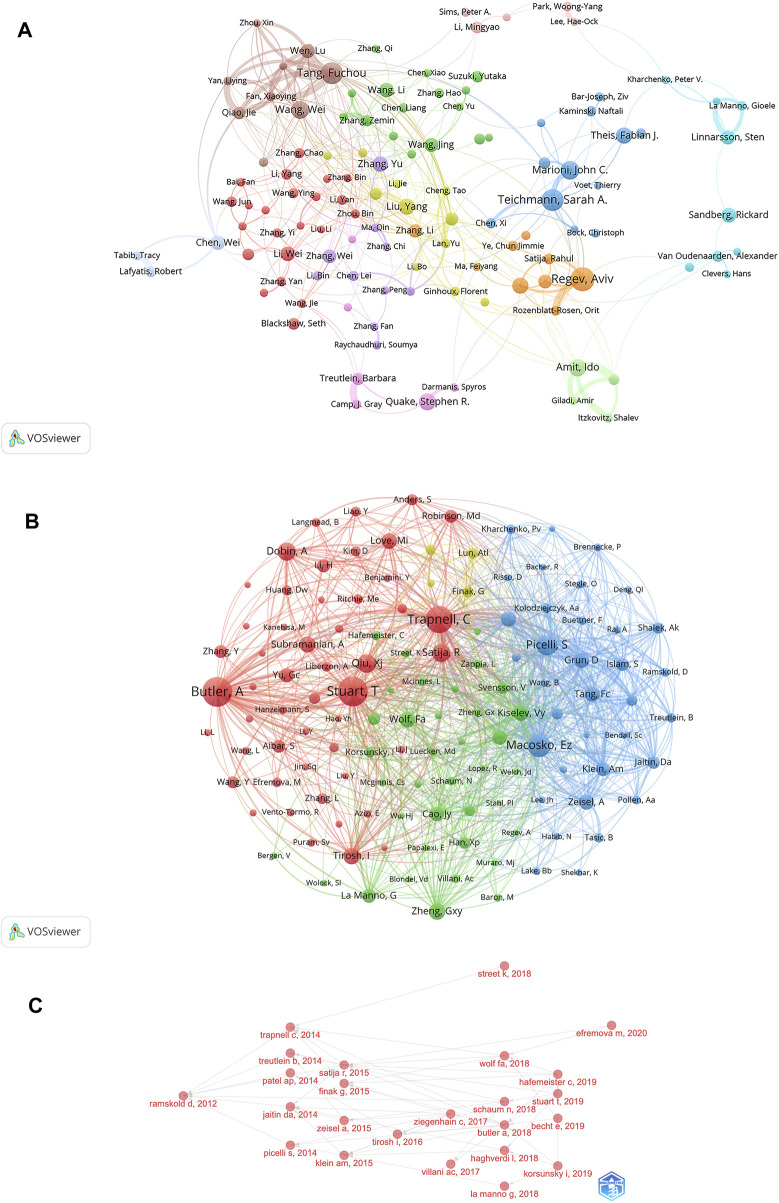
Analyses of single-cell relevant authors. **(A)** Visualization of authors’ collaborative networks in VOSviewer. **(B)** An analysis of the VOSviewer collaborative network visualization of author citations. **(C)** Analysis of citation relationships among SCS related authors.

Identification of the most prolific authors according to the number of publications and co-citations in the field of SCS could provide insights into hotspots of research. The top ten most prolific authors produced 447 papers, representing 4.50% of all publications in the field (Table 3). Regev A was identified as the most prolific author, having produced 61 papers (0.61% of the total publications). With 55 reports (0.55% of the total), Teichmann SA, ranked second in terms of quantity of publications. The term “co-cited authors” refers to a situation where two or more authors are cited together in one or more subsequent works. The two most cited among the top ten authors were Regev A and Teichmann SA, who were collectively cited in over 2000 articles (Table 3). This was followed by Stuart T (1,928 co-citations), Macosko EZ (1,527 co-citations), and Picelli S (1,303 co-citations). We conducted further analysis of co-cited authors with the aid of VOSviewer (Figure 5A). The total link strength is the sum of the link strength between a node (such as a journal or scholar) and other nodes, providing a measurement of the degree of relatedness between nodes A larger total link strength value indicates that the node is more closely or strongly connected to other nodes, which could be used to assess the influence of an academic journal or scholar and size of a collaborative network. As shown in Figure 5A, the strongest link strength was observed for the authors Butler A, Stuart T, and Trapnell C, demonstrating their significant influence in the field of SCS.

**TABLE 3:** Top 10 authors and co-cited authors related to stem cells in stroke.

Rank	Authors	Count (%)	Total link strength	Authors	Co-citations	Total link strength
1	Regev A	61(0.61%)	51	Butler A	2,201	18,080
2	Teichmann SA	55(0.55%)	43	Stuart T	2,167	17,396
3	Tang F	54(0.54%)	128	Trapnell C	1928	20,534
4	Marioni JC	44(0.44%)	24	Macosko EZ	1,527	18,130
5	Wang W	42(0.42%)	38	Picelli S	1,303	16,108
6	Amit I	41(0.41%)	55	Qiu X	1,117	11,887
7	Quake SR	39(0.39%)	15	Dobin A	1,105	9,986
8	Liu Y	37(0.37%)	27	Satija R	1,085	10,846
9	Shalek AK	37(0.37%)	23	Tirosh I	974	10,880
10	Theis FJ	37(0.37%)	9	Zheng GX	967	10,954

VOSviewer provided critical insights into the collaborations of authors involved in research on SCS (Figure 5B). The various colors of the nodes reflect authors in different clusters and node diameter signifies the frequency of occurrence. According to the co-cited author network diagram, authors were roughly divided into four categories. The collaborative network in our study was centered on Regev A and Teichmann SA. Our results showed associations of Regev A with Rozenblatt-Rosen O, Satija R, Teichmann SA, and Marioni JC, while Tang F worked closely with Qiao J, Fan X, Yan L, and Chen X had active ongoing collaborations.

The histogram depicts the distribution of SCS-related authors from the top 20 countries (Supplementary Figure S1A). American authors ranked first in terms of the number of comprehensive articles in this field. The percentage of single-country publications (SCP, green) was greater than that of multinational joint publications (MJP, red). USA was followed by China, Britain, Germany, Japan, Canada, Australia, Sweden, Switzerland, and France.

The development of research hotspots in the SCS field could be predicted by sorting the published papers and authors in relation to time, and analyzing the citation relationships among relevant authors (Figure 5C; Supplementary Figure S1B). Ramskold D published a Smart-Seq protocol in *OncoTargets and Therapy* in 2012. In 2014, Trapnell C, Treutlein B et al. applied the above protocol. On this basis, in 2015, Satijia R, Finak G, and co-workers published an article on Model-based Analysis of Single-cell Transcriptomics. After 2017, cross-referencing by authors in the field became more frequent.

### 3.5 Analyses of academic journals and co-citations

We identified several periodicals with published articles related to SCS, including *Cell*, *Science*, *Nature,* and other well-known journals. The Vosviewer tool was employed to analyze previously published papers and identify journals with high publication rates and impact as well as understand the scholarly impact of these journals within domains associated with SCS. The top three journals that published the largest number of papers were *Nature Communications*, *Frontiers in Immunology*, and *Cell Reports*, with 539, 336, and 255 articles respectively. Notably, *Nature Communications* had the highest impact factor and number of overall publications among the journals examined. Through analysis of the citations, key journals could be located. *Nature* (28,894) was the most highly co-cited among the top ten journals, clearly indicating its considerable influence in research communications on SCS. The Journal Citation Reports (JCR) quartiles were sorted according to the impact factors of various fields in the current year and subsequently categorized into four distinct sections designated Q1, Q2, Q3, and Q4. Q1 signifies the top 25% of journals in the impact factor classification by discipline, followed by Q2 (top 25%–50% of journals), Q3 (top 50%–75% of journals), and Q4 (journals below 75%). Table 4 displays the top ten journals ranked based on production. Clearly, 80% of the top ten journals that published the most papers belonged to Q1 and the remaining 20% to Q2. Moreover, the top ten journals presented in Table 4 belonged exclusively to Q1.

**TABLE 4:** Top 10 journals and co-cited journals related to single-cell sequencing.

Rank	Journal	Count (%)	IF(JCR 2021)	JCR quatile	Co-cited-journal	Citations	IF(JCR 2021)	JCR quatile
1	Nature Communications	539 (5.43%)	17.694	Q1	Nature	28,894	69.504	Q1
2	Frontiers In Immunology	336 (3.38%)	8.787	Q1	Cell	27,508	66.85	Q1
3	Cell Reports	255 (2.57%)	9.995	Q1	Science	19,524	63.832	Q1
4	Bioinformatics	231 (2.33%)	6.931	Q1	Nat Commun	16,477	17.694	Q1
5	Scientific Reports	192 (1.93%)	4.997	Q1	P Natl Acad Sci Usa	15,800	12.779	Q1
6	Elife	174 (1.75%)	8.713	Q1	Nat Methods	15,380	47.99	Q1
7	Genome Biology	167 (1.68%)	18.01	Q1	Nat Biotechnol	13,647	68.164	Q1
8	Frontiers In Cell And Developmental Biology	164 (1.65%)	6.081	Q1	Genome Biol	11,049	18.01	Q1
9	Frontiers In Genetics	164 (1.65%)	4.772	Q1	Bioinformatics	10,810	6.931	Q1
10	Proceedings Of The National Academy Of Sciences Of The United States	163 (1.64%)	12.779	Q1	Nucleic Acids Res	9,375	19.16	Q1

**FIGURE 6:**
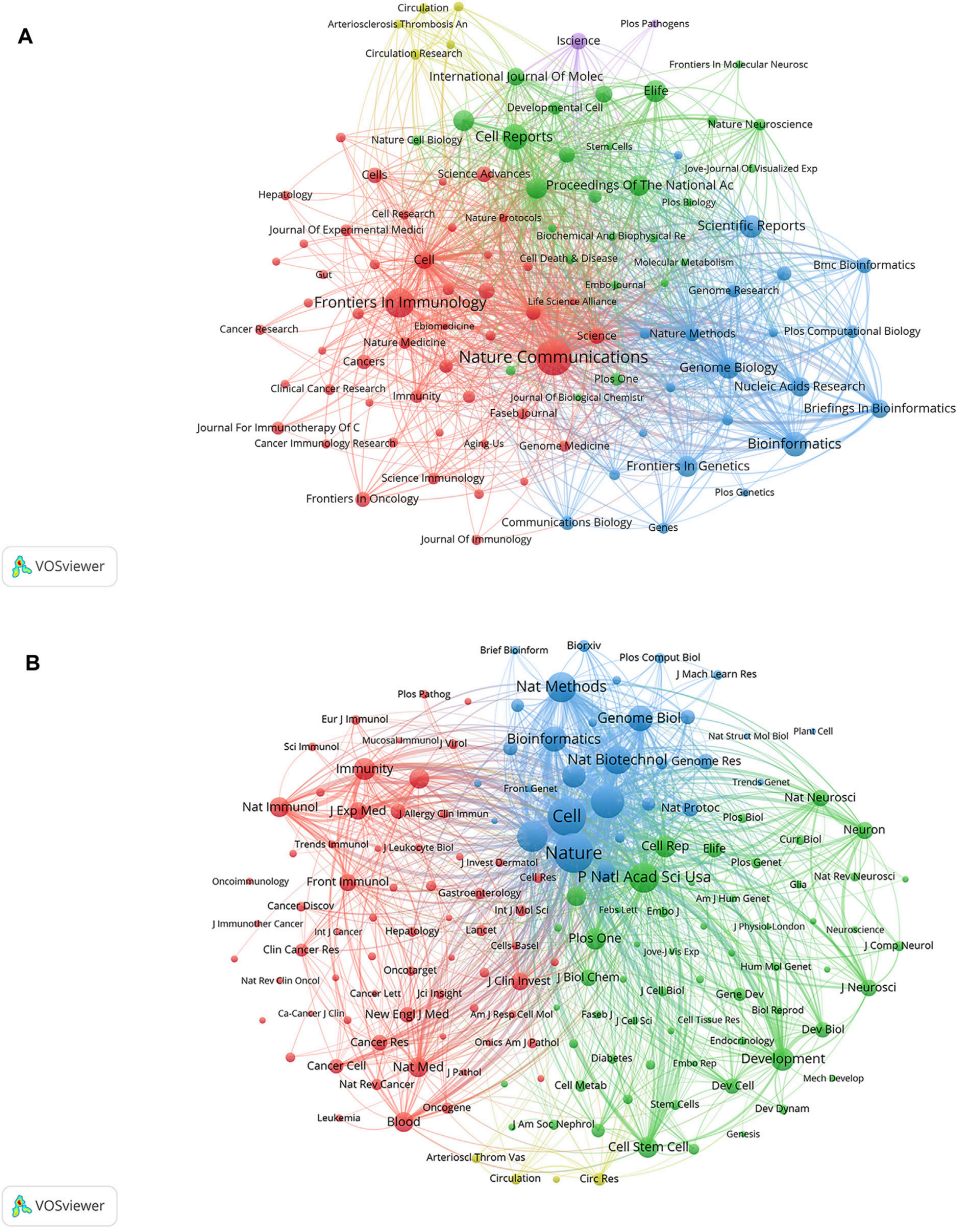
Analyses of single-cell relevant journals and co-cited academic journals. **(A)** Journal clustering analyses related to SCS. Each circle symbolizes a journal, and the diameter varies based on factors such as the number of citations and strength of the relationship. Additionally, the circle’s color indicates the cluster to which it belongs, with various colors indicating different clusters. **(B)** Co-cited journals related to SCS.

Data from cluster analysis of journals with articles related to SCS are presented in Figure 6A. Each circle indicates a journal and the diameter is variable depending on multiple factors, such as strength of the relationship and quantity of citations. Moreover, each cluster is indicated by a different hue on the circle according to the cluster to which it is assigned. Overall, clustering in this study was divided into five types. The red cluster included studies pertaining to immunology and biological sciences (*Nature Communications*, *Frontiers in Immunology*), the blue cluster included studies on bioinformatics and genome biology (*Scientific Reports*, *Bioinformatics*, *Genome Biology*), the green cluster included publications focusing primarily on cell biology (*Cell Reports*, *Proceedings of the National Academy of Sciences of the United States*, *eLife*), the yellow cluster included studies on the circulatory system (*Circulation Research*, *Arteriosclerosis Thrombosis and Vascular Biology*, and *Circulation*), and the purple cluster included studies on the life sciences (*iScience*).

According to the quantity of co-citations, academic periodicals were categorized into four clusters (depicted in Figure 6B), indicating a tendency to follow similar research trajectories. The red cluster mainly represents immunology (*Immunity*, *Nature Immunology*, *Frontiers in Immunology*, and others), the green cluster cell biology (*Proceedings Of The National Academy of Science of the United States*, *Development*, *Cell Reports*, *Cell Stem Cell*), the blue cluster cytology (*Cell*, *Nature*, *Nature Methods*), and the yellow cluster the cardiovascular system (*Arteriosclerosis Thrombosis And Vascular Biology, Circulation, Circulation Research*).

In addition, we conducted a dual-map overlay study of journals using VOSviewer. Labels on the right of Supplementary Figure S2 represent the referenced journals while those on the left symbolize SCS-related cited journals. Curves represent the citation line. The length of the vertical axis is proportional to the number of published papers and the elliptical horizontal axis to the number of authors. We identified three primary citation paths (yellow, green, and red), which indicated that researchers principally cited publications from molecular biology, immunology, dermatology, surgery, and clinical periodicals.

### 3.6 Analyses of keywords

**FIGURE 7:**
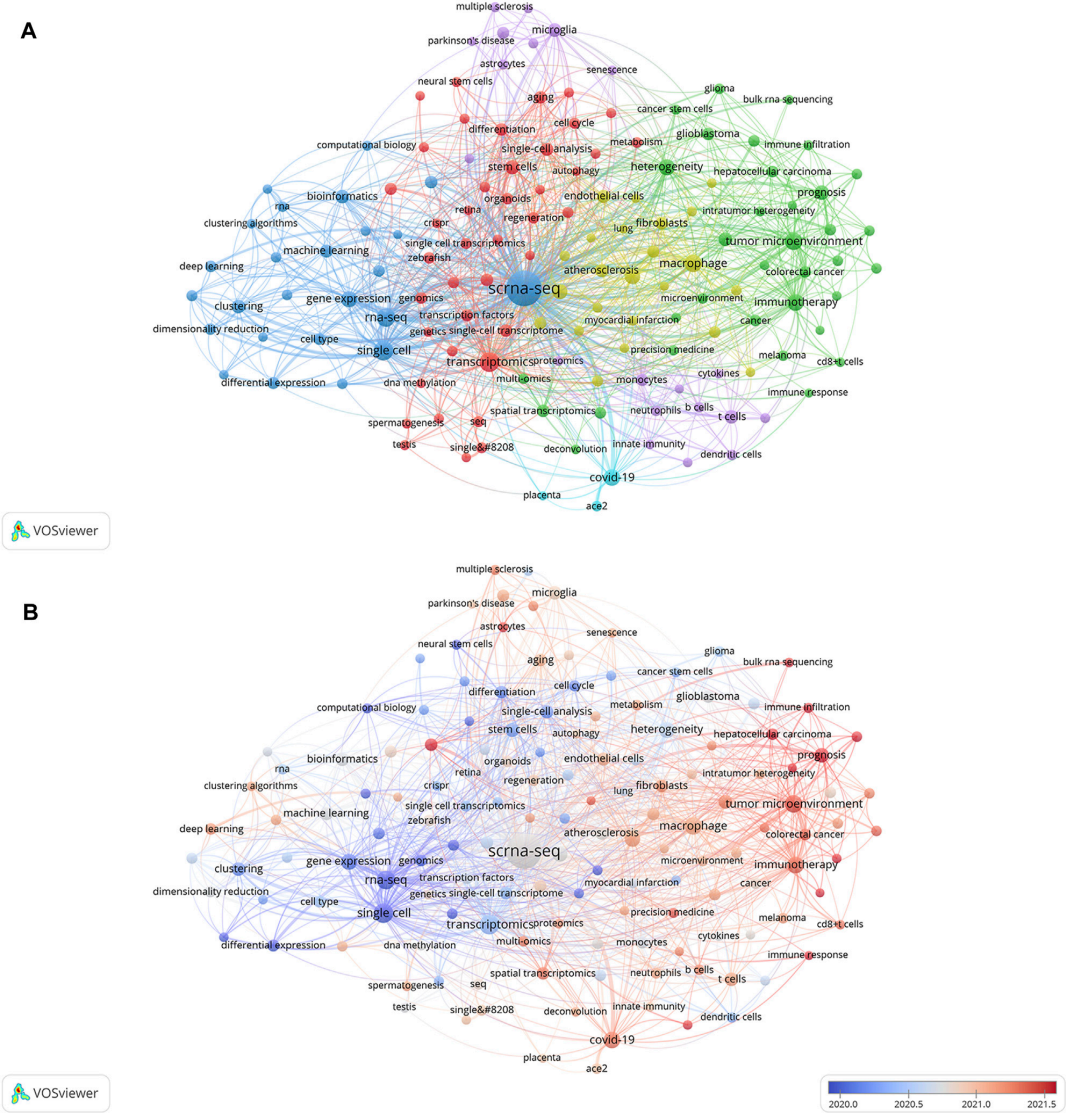
Analyses of single-cell relevant keywords. **(A)** Clustering analysis of keywords. **(B)** Keywords heat map on SCS.

Relevant manuscripts can be successfully identified by researchers through the use of keywords. Analysis of keywords in papers assists in highlighting popular topics and current scientific issues. The top 20 terms with the greatest overall link strength and frequency in our study are shown in Table 5. In addition to “scrna-sep” (2,461 times) and “single-cell” (300), the keyword “transcriptomics” appeared frequently (299), followed by “rna-sep” (253) and “tumor microenvironment” (206). Research on SCS is particularly focused on fields associated with transcriptomics. The keyword clustering function of VOSviewer serves to classify and summarize hotspots in a certain research area during a specific time frame to identify study hotspots in a specific field. SCS-related keywords in the literature were grouped using VOSviewer. As shown in Figure 7A, each of the labels and circles represents a separate unit, and each colored unit forms a unique cluster. Keywords were grouped into five clusters: application of SCS in tumors (green), introduction and classification of SCS (navy blue), studies on the intracellular mechanisms of SCS (red), studies on SCS in the immune system (purple), SCS in the cardiovascular system (yellow), and studies on SCS in COVID-19 (light blue).

**TABLE 5:** Top 20 keywords in terms of frequency of occurrence and the corresponding total link strength.

Rank	Keyword	Occurrences	Total link strength
1	scrna-seq	2,461	2,811
2	single-cell	300	578
3	transcriptomics	299	609
4	rna-seq	253	442
5	tumor microenvironment	206	368
6	macrophage	191	375
7	covid-19	164	234
8	immunotherapy	158	302
9	gene expression	146	268
10	heterogeneity	140	290
11	inflammation	125	240
12	mouse	118	192
13	prognosis	99	200
14	stem cells	89	158
15	clustering	85	158
16	biomarker	84	144
17	microglia	83	172
18	bioinformatics	82	175
19	t cells	81	129
20	machine learning	73	147

Keyword analysis elucidated the popularity and patterns of research in the realm of SCS. A heat map of SCS keywords was generated to establish the frequency of keywords used (Figure 7B). In the figure, red segments signify more recent and frequent keyword appearances whereas blue sections represent keywords from relatively early studies. “Tumor microenvironment”, “immunotherapy”, “hepatocellular carcinoma”, “prognosis”, and “COVID-19” were identified as topics of active research in the last few years, providing an overview of the current research hotspots.

### 3.7 Analyses of cited references

**FIGURE 8:**
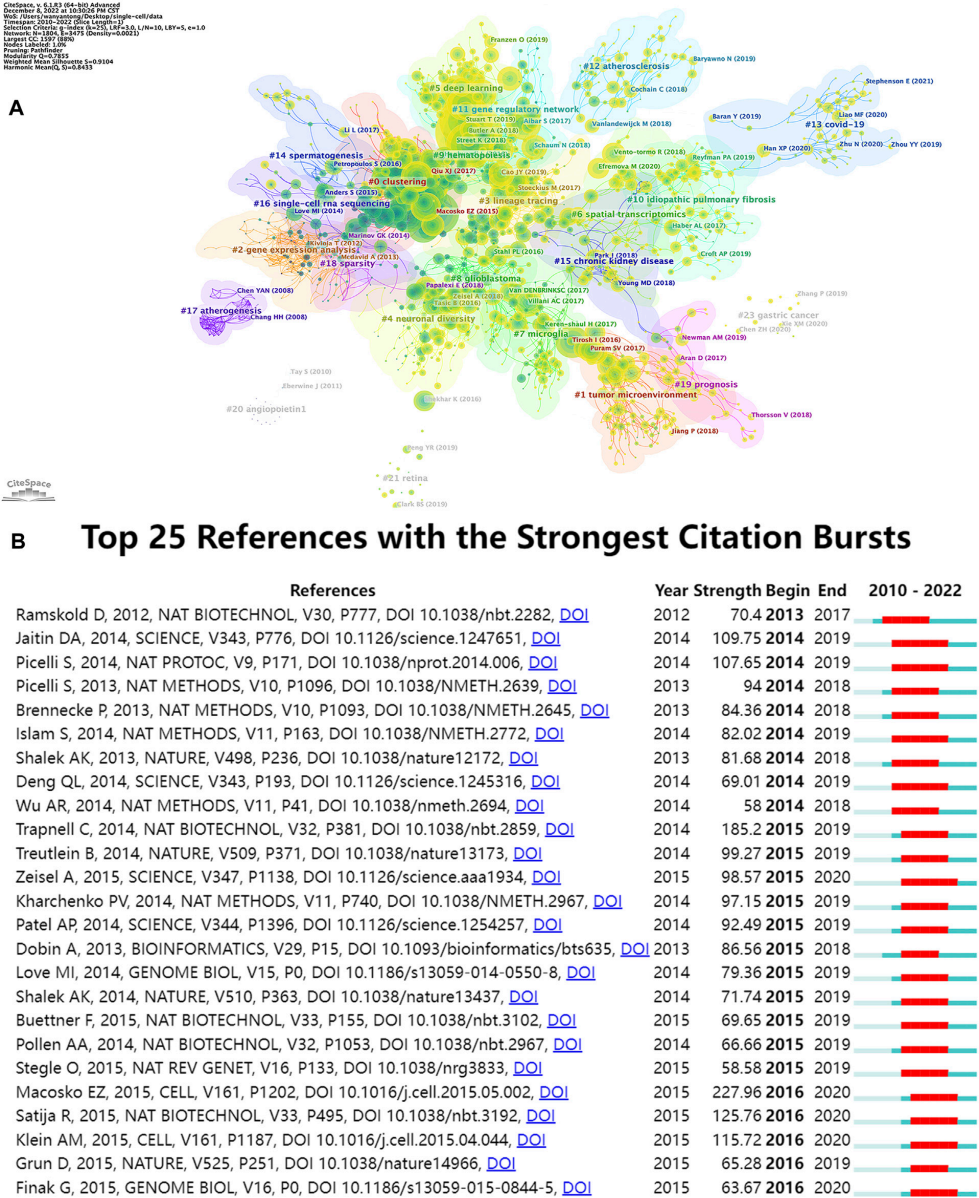
Analyses of single-cell relevant cited references. **(A)** Cluster view of references in SCS research. **(B)** The top 25 references that had the most significant bursts of citations.

The top 15 most frequently cited papers among the 9,929 manuscripts included for study are displayed in Table 6. The most cited publication (3,846 citations) presents a novel analytical method of integrating scRNA-seq datasets, entitled “Integrating Single-cell Transcriptome Data Across Various Circumstances, Technologies, and Species” (Butler et al., 2018). The next most common reference was “Comprehensive Integration of Single-Cell Data” (3,801 citations). This article offers a method for integrating single-cell observations by “anchoring” various data sets. The protocol is effective across scRNA-seq technologies as well as various other modalities (Stuart et al., 2019). The third was a publication by Patel AP (1,378 citations) that revealed hitherto underappreciated diversity in various regulatory processes crucial to the biology, prognosis, and treatment of glioblastoma (Patel et al., 2014). A map classifying references into 23 groups using the cluster analysis function of Citespace is presented in Figure 8A. The diameter of a circle reflects the number of citations in the paper. The calculated weighted mean silhouette was 0.9104 while the modularity Q value was 0.7855, signifying high clustering structure stability and credibility. The map highlighted the newest research trends. The largest grouping was “clustering” (cluster #0), followed by “tumor microenvironment” (cluster #1), “gene expression analysis” (cluster #2), and “lineage tracing” (cluster #3). Other notable clusters included “neuronal diversity”, “deep learning”, and “spatial transcriptomics”. The top 25 references with the most significant citation bursts are displayed in Figure 8B. Citation bursts indicate a sudden and increasingly rapid rise in the number of citations. A reference with a strong citation burst represents an article that is frequently cited during a certain period of time. The first citation bursts occurred in 2012. The relevant study (Ramskold et al., 2012) described the possibility of genome-wide transcriptome analysis in individual cells. Notably, a report entitled “Highly parallel genome-wide expression profiling of individual cells using nanoliter droplets” by Macosko EZ and co-workers (Macosko et al., 2015) published in the journal *Cell* in 2015 was highlighted as the article with the greatest burst (strength = 227.96) over a duration of 5 years until 2020. According to the data, 2015 and 2014 were the years that had the most recent citation bursts, occurring 11 and 8 times respectively, implying that the linked research boom was caused by the high-burst publications in these 2 years. Scholars are particularly interested in research involving scRNA-seq, as evident from the multiple citation bursts in this direction by 2020.

**TABLE 6:** Top 15 cited references related to single-cell sequencing.

Rank	Author	Article title	Source title	Cited	Year	DOI
1	Butler A et al	Integrating single-cell transcriptomic data across different conditions, technologies, and species	NATURE BIOTECHNOLOGY	3,846	2018	10.1038/nbt.4096
2	Stuart T et al	Comprehensive Integration of Single-Cell Data	CELL	3,801	2019	10.1016/j.cell. 2019.05.031
3	Patel AP et al	Single-cell RNA-seq highlights intratumoral heterogeneity in primary glioblastoma	SCIENCE	2,326	2014	10.1126/science.1254257
4	Trapnell C et al	The dynamics and regulators of cell fate decisions are revealed by pseudotemporal ordering of single-cells	NATURE BIOTECHNOLOGY	2,260	2014	10.1038/nbt.2859
5	Tirosh I et al	Dissecting the multicellular ecosystem of metastatic melanoma by single-cell RNA-seq	SCIENCE	1900	2016	10.1126/science.aad0501
6	Satija R et al	Spatial reconstruction of single-cell gene expression data	NATURE BIOTECHNOLOGY	1857	2015	10.1038/nbt.3192
7	Picelli S et al	Full-length RNA-seq from single-cells using Smart-seq2	NATURE PROTOCOLS	1845	2014	10.1038/nprot. 2014.006
8	Klein AM et al	Droplet Barcoding for Single-Cell Transcriptomics Applied to Embryonic Stem Cells	CELL	1737	2015	10.1016/j.cell. 2015.04.044
9	Zeisel A et al	Cell types in the mouse cortex and hippocampus revealed by single-cell RNA-seq	SCIENCE	1,653	2015	10.1126/science.aaa1934
10	Becht E et al	Dimensionality reduction for visualizing single-cell data using UMAP	NATURE BIOTECHNOLOGY	1,466	2019	10.1038/nbt.4314
11	Sungnak W et al	SARS-CoV-2 entry factors are highly expressed in nasal epithelial cells together with innate immune genes	NATURE MEDICINE	1,336	2020	10.1038/s41591-020-0868-6
12	Wolf FA et al	SCANPY: large-scale single-cell gene expression data analysis	GENOME BIOLOGY	1,301	2018	10.1186/s13059-017-1,382-0
13	Zou X et al	Single-cell RNA-seq data analysis on the receptor ACE2 expression reveals the potential risk of different human organs vulnerable to 2019-nCoV infection	FRONTIERS OF MEDICINE	1,245	2020	10.1007/s11684-020-0754-0
14	Ziegler C et al	SARS-CoV-2 Receptor ACE2 Is an Interferon-Stimulated Gene in Human Airway Epithelial Cells and Is Detected in Specific Cell Subsets across Tissues	CELL	1,235	2020	10.1016/j.cell. 2020.04.035
15	Liao MF et al	Single-cell landscape of bronchoalveolar immune cells in patients with COVID-19	NATURE MEDICINE	1,178	2020	10.1038/s41591-020-0901-9

### 3.8 Analyses of subject area

Subject area analyses of SCS literature were conducted using CiteSpace (presented in Supplementary Figure S3). The most cited academic field was “*CELL BIOLOGY*”, followed by “*BIOCHEMISTRY and MOLECULAR BIOLOGY*”, “*GENETICS and HEREDITY*”, and “*MULTIDISCIPLINARY SCIENCES*.” The purple circles surrounding these disciplines reflect their importance in this field. In particular, “*CELL BIOLOGY*”, “*BIOCHEMISTRY and MOLECULAR BIOLOGY*”, “*GENETICS and HEREDITY*”, “*IMMUNOLOGY*”, “*CELL BIOLOGY*”, “*ONCOLOGY*”, and “*NEUROSCIENCES”* are denoted by purple circles, indicating a greater influence of these disciplines within the field of SCS.

## 4 Discussion

### 4.1 Global research trends of SCS

This research focused on 9,929 publications related to SCS in the WOSCC, spanning 1 January 2010, until 3 December 2022. Figure 2 displays the annual trend of studies published on SCS. The first article, published by Tang F of Peking University in 2009 (Stuart et al., 2019), marked the beginning of advancements in SCS. In 2015, two teams from Harvard University combined micro-low fluid technology with single-cell RNA-seq to develop drop-seq (Macosko et al., 2015) and in-drop (Klein et al., 2015), respectively, which were published in the same issue of *Cell*. These two techniques could facilitate simultaneous sequencing of all genes and trace the cell of origin for each gene. Their emergence facilitated analysis of gene expression in thousands of single cells in a rapid and low-cost manner. Since then, rapid advances have been made in the field of single-cell sequencing technology. In 2018, single-cell sequencing was named one of the top ten scientific breakthrough technologies by *Science*, and in 2019, awarded “Technology of the Year” in the field of life sciences by *Nature Methods*. The field of SCS has seen progressive advances over the years and the number of related papers continues to increase. From 2019 to 2021, the number of publications increased significantly, reaching a peak in 2022. The overall findings indicate that SCS technology has become increasingly popular over the past few years and is undergoing a rapid developmental phase.

The quantity of papers and total link strength were the two most significant metrics in nation/region analyses. Similarly, the level of cooperation between nations or regions was reflected by total link strength. The United States had the highest number of publications, citation frequency and total link strength, followed by China (second in quantity of publications, third in citation frequency and fourth in total link strength). Five of the top ten institutions were from the Chinese mainland while three were American, as shown in Tables 1, 2. Based on the above data, China and the US were identified as the two nations that have made the greatest contributions to the field of SCS to date. Other countries with significant contributions to the advancement of SCS include England, Germany and, Switzerland.

The highest co-citation frequency was for Butler A, highlighted the significant contribution of this researcher to SCS-related fields (Table 3; Figure 5). In 2019, Butler A et al. (Stuart et al., 2019) published an article introducing a SCS data integration system that could successfully transfer information between single-cell transcriptome, proteome, epigenome, and spatial information datasets. The scheme was based on thorough, updated statistical models. At that time, sequencing costs were still high and the sequencing process was complex. The data integration method described in the report undoubtedly provided a powerful tool for full mining and joint analysis of biological targets from existing and emerging data. Additionally, this publication had the highest quantity of cited articles, with 3,801 citations. The study was conducted under the guidance of Satija R, the corresponding author of this paper. Regev A, was the most prolific, followed by Teichmann SA, Tang F, and Marioni JC. These researchers are pioneers in this field and have made significant contributions to numerous publications. Notably, in 2009, Tang F published a study on single-cell mRNA sequencing, which pioneered SCS research. This work revolutionized the field and introduced the era of single-cell gene expression analysis. Satija R and co-workers have developed a software known as Seurat (Delorey et al., 2021), which locates individual cells in 3D spatial models of tissues and identifies cell subtypes. This technique is valuable for exploring the organizational origin of each transcriptome. Regev A made important contributions in the field of single-cell sequencing which has significantly advanced our understanding of cellular diversity and biological complexity.

Single-cell RNA sequencing technology was initially developed by Regev A. Over the years, her group has been involved in the development of a number of advanced single-cell RNA sequencing technologies, such as drop-seq and inDrop (Macosko et al., 2015), which facilitate high-throughput single-cell gene expression analysis using microdroplet technology. These methods have provided effective tools to explore gene expression patterns in individual cells, revealing the diversity of cell types and states. Moreover, Regev A is involved in studies on cell type that highlight differences in gene regulation between cell types. Using SCS, the research group has successfully mapped cell types within multiple tissues and organs, and conducted detailed analyses of transcriptional regulatory networks between cell types (Kalluri et al., 2019). These findings provide a deeper understanding of cellular functions and developmental processes. In addition, Regev A has focused on uncovering the mechanisms underlying the heterogeneity of tumor cells. With the aid of SCS technology, her team has evaluated gene expression and mutational patterns of different cell subsets within tumors, revealing mechanisms of tumor evolution and drug resistance with important implications for cancer therapy and individualized treatment regimens (Patel et al., 2014; Tirosh et al., 2016; Venteicher et al., 2017). The collective contributions of these researchers to the field of SCS have provided critical breakthroughs, improving our understanding of cellular diversity, biological complexity and related diseases (Macosko et al., 2015).

*Nature Communications*, *Frontiers in Immunology* and *Cell Reports* are the top three periodicals in terms of quantity of related reports, with 539, 336, and 255 publications, respectively (Table 4; Figure 6). Among the ten leading periodicals, 80% were classified as Q1 and 20% as Q2. *Nature* ranked first with 28,894 citations, followed by *Cell* with 27,508 citations and *Science* with 19,524 citations. All the top ten cited journals were from Q1. A quarter of the top ten journals had an impact factor (IF) greater than 60 based on the journal citation reports, including *Nature*, *Cell*, *Science*, and *Nature Biotechnology*. *Nature Methods* had an IF value between 40 and 50, while four other journals (*Nature Communications*, *Proceedings of the National Academy of Sciences of the United States*, *Genome Biology*, and *Nucleic Acids Research*) had IF values between 10 and 20. *Bioinformatics* had an IF value between 5 and 10. The overall results indicate that SCS studies are generally of high quality. Furthermore, the published research was predominantly focused on cytology, biology, and immunology.

### 4.2 Advances in SCS

With the development of technology and expansion of application areas, single-cell sequencing has triggered many novel technological advances. This article mainly focuses on three aspects: multi-modal single-cell analysis, data processing, and new technology development.

#### 4.2.1 Application of multimodal single-cell analysis

Single-cell multimodal omics is a method that combines multiple single-cell sequencing technologies to obtain comprehensive and accurate cell information (Yu et al., 2023). This technique allows simultaneous analysis of the multimodal molecular attributes of gene expression, chromatin accessibility, and protein abundance at the global level of individual cells, enabling researchers to clarify cell heterogeneity and understand the fine cellular states, currently a cutting-edge field in genomics research (Di et al., 2020; Zhu et al., 2020). With the continuous improvement of multimodal single-cell analysis methods, this technology has been progressively applied in the disciplines of cancer research (Herrera et al., 2021; Koya et al., 2021), vaccination (de Assis et al., 2023; Sparks et al., 2023), and other related fields (Jang et al., 2023; Mullin et al., 2023).

#### 4.2.2 Improvement in handling and analysis of diverse sample data

In recent years, data analysis methods for single-cell sequencing have progressed rapidly. An increasing number of data processing and analysis software tools have been developed, greatly reducing the barriers to processing and analysis of single-cell sequencing data. The main purpose of single-cell latent variable models is extraction of information from large-scale high-dimensional data to reveal the underlying characteristics and biological states of cells. Depending on the nature of the latent variables and structure of data, single-cell latent variable models can be divided into different methods and algorithms, including Latent Semantic Analysis (Lozoya et al., 2020), Latent Dirichlet Allocation (Lou et al., 2023), Factor Analysis (Buettner et al., 2017), and Variational Autoencoder (Rashid et al., 2021). Compared with the traditional algorithm based on population averages, the single-cell latent variable model is more effective in accurately describing and resolving the heterogeneity between cells (Liu et al., 2021). The model is able to classify cells into different subpopulations or types, and infer characteristics and states (Liu et al., 2019), such as cell type and developmental status. Moreover, single-cell latent variable models have been utilized to identify previously unknown cell subsets by mining structural patterns in low-dimensional latent space, extract latent variables from large-scale single-cell data and cluster cells into subsets with similar characteristics, leading to the discovery of new cell types and functions (Liu et al., 2019). Conos is a method that relies on multiple trusted sample mappings to construct a global graph connecting all measured cells. The graph can effectively identify recurrent cell clusters and propagate information between datasets in multi-sample or atlas-scale collections (Barkas et al., 2019). The basic function of Conos is to construct a relationship network between cells by calculating their similarity and overlap via six steps: data preprocessing, construction of a similarity matrix, overlap calculation, construction of a cell network, cell clustering, visualization, and analysis (Barkas et al., 2019), providing a joint analysis of heterogeneous single-cell RNA-seq dataset collections. In contrast to traditional single-cell sequencing analysis algorithms, Conos calculates the overlap, representing genes that are commonly expressed between cells, which facilitates more accurate discrimination of cell subsets. Conos also has the advantageous ability to process large-scale single-cell sequencing data and visualize cell networks. The hierarchical Poisson factorization uses Bayesian inference to model single-cell sequencing data, which can be adapted to various data types and characteristics (Gopalan et al., 2014). Compared with linear assumption of data in the traditional algorithm, this algorithm can better capture nonlinear data relationships and improve the description ability of the model. In addition, a penalty term can be introduced to reduce the complexity of the model and improve its robustness to noise, which effectively removes noise and outliers from the data, and improves the accuracy and stability of the model (Levitin et al., 2019). Moreover, the proposed algorithm can extract the relationship between potential biological features and gene expression distribution from single-cell sequencing data, presenting a powerful processing tool that effectively mines hidden information within the datasets and provides critical support for biological research. These novel single-cell sequencing data analysis methods provide improved tools and procedures, allowing accurate analysis and identification of different cell subtypes, and in-depth characterization of the properties of individual cells.

#### 4.2.3 Development of new single-cell sequencing technologies

In recent years, various new single-cell sequencing technologies have been developed, including single-cell methylation sequencing, single-cell ATAC sequencing, and single-cell proteomics sequencing. Single-cell ATAC-seq can effectively reveal the accessibility of chromatin regions using the *in situ* transposase technique (Liu et al., 2019). Recent studies using ATAC-seq technology identified major and subclass-specific cell types and cis-regulatory elements in the mouse cerebral cortex, and further analyzed the heterogeneity of chromatin accessibility (Xu et al., 2022). SPLitseq (split-pool ligation-based transcriptome sequencing) is a low-cost scRNAseq method based on split-pool single-cell sequencing technology, which can achieve transcriptional analysis of thousands of fixed cells or nuclei in a single experiment (Rosenberg et al., 2018). To meet the needs of bacterial scRNA-seq, researchers have developed a single-cell transcriptome sequencing protocol for prokaryotic cells, which uses the split-pool method to label individual bacterial cells and complete single-cell RNA sequencing of prokaryotic cells (Blattman et al., 2020; Kuchina et al., 2021). The emergence of these novel technologies is expected to facilitate comprehensive and accurate analysis of cell features, and expand the applications of single-cell sequencing technology in basic medicine and clinical diagnosis.

### 4.3 Hotspots and frontiers

Elucidation of the cutting-edge and trending issues in SCS was further achieved using keyword analysis. The primary search terms in previous investigations were “scrna-seq,” “single-cell,” “transcriptomics”, “rna-seq”, “tumor microenvironment”, “macrophage”, “COVID-19”, “immunotherapy”, “gene expression”, and “heterogeneity” (Table 5), representing popular topics of SCS research. A heat map of these keywords revealed “tumor microenvironment”, “immunotherapy”, “hepatocellular carcinoma”, “prognosis”, and “COVID-19” as research hotspots in this discipline (Figure 8B). Moreover, the majority of research was conducted in the fields of oncology, neuroscience, and developmental biology (Supplementary Figure S3).

#### 4.3.1 SCS and oncology

The relationship between SCS and tumors is currently an area of increasing interest. In the field of oncology, significant progress in research on the origin, development, and treatment of tumors has been made with SCS. Intra-tumor heterogeneity is one of the main factors underlying poor therapeutic effects and recurrence. Using SCS, researchers can gain insights into cellular heterogeneity within tumors, leading to improved understanding of the mechanisms underlying tumor development and treatment resistance. For instance, SCS technology has provided key insights into the heterogeneity of tumor cells in breast cancer. Different subclones show distinct expression and mutation profiles, which could be used to inform novel therapeutic strategies for breast cancer (Thakur et al., 2023). SCS is employed by researchers to understand the type, number, and functional status of tumor immune cells. Analysis of immune cells in melanoma patients has shown that the diversity of tumor-infiltrating T cells is closely related to survival, providing an important basis for individualized immunotherapy (Li et al., 2022; Vasudevan et al., 2023). Resistance of tumor cells to chemotherapeutic drugs is a considerable challenge in clinical treatment. SCS has the ability to uncover the internal drug resistance mechanisms of tumor cells, thus providing a basis for individualized drug therapy (Dai et al., 2020). For example, an earlier study using SCS to characterize chemotherapy drug-resistant cells in colon cancer patients showed specific gene expression patterns in these cells, which could serve as an important indicator for predicting drug resistance and developing new therapeutic strategies (Wang et al., 2022). In addition, the tumor microenvironment has a significant impact on tumor development and, consequently, therapeutic efficacy. In this regard, interactions between tumor cells and their surrounding cells can be effectively distinguished using SCS (Tian and Li, 2022). Analysis of the tumor microenvironment of pancreatic cancer patients with SCS revealed that interactions between tumor and pancreatic stellate cells play an important role in cancer growth and metastasis, providing new ideas for designing therapeutic strategies targeting the tumor microenvironment (Zhang et al., 2021). Recently, pro-nociceptin and leukocyte-associated immunoglobulin-like receptor 2 were identified as biomarkers for assessment of immune infiltration in cholangiocarcinoma using a combination of batch sequencing and single-cell sequencing (Chen et al., 2021).

In summary, the utility of SCS in oncology involves numerous aspects, such as tumor heterogeneity, evolution, tumor immunology, drug resistance, and the microenvironment. However, multiomics technology is often restricted by flux limitations, artificial operation, and other limitations, which inhibit its widespread adoption. Furthermore, the high cost makes it challenging for smaller laboratories to participate in technological advancements and improvements. As a result, the direction of SCS is moving towards achieving automated, high-throughput, and cost-effective approaches. As SCS technology continues to develop, its impact on cancer research is expected to increase, potentially leading to significant contributions to the development of tumor precision medicine.

#### 4.3.2 SCS and neuroscience

The nervous system is the most dominant and complex system in the human body, exhibiting a high degree of heterogeneity. Clarification of the complexities that arise from this system can aid in the prediction, diagnosis, and treatment of neurological diseases. SCS has been effectively applied to determine the molecular characteristics, developmental processes, and synaptic connections of different cell types in the nervous system. Research on the use of SCS in understanding the mechanisms underlying various nervous system diseases is progressively gaining importance. For instance, a study on Parkinson’s disease used single-cell RNA sequencing to reveal the molecular signatures and abnormally expressed genes of dopaminergic neurons (Smajic et al., 2022). Based on single-cell sequencing of dopaminergic neurons in a population of PD patients and healthy controls, the investigators identified disease-related genes that were abnormally expressed, which provided important clues for understanding the pathogenesis of PD and identifying new treatments (Duan et al., 2021). Moreover, SCS has provided deeper insights into nervous system functions and disease mechanisms. Another study conducted single-cell analysis of the cortex and hippocampus. Earlier experiments have shown that hippocampus may play a role in the pathogenesis of autoimmune demyelination through immunosuppression and inflammation regulation of the central nervous system. Finally, SCS was employed to examine neuronal cell types and establish connectivity maps. Single-cell transcriptome sequencing has been applied to delineate the molecular profile and heterogeneity of the hippocampus in type 2 diabetic mice. By focusing on microglia subsets, pathological changes of hippocampal injury mediated by inflammation and oxidative stress in mice were revealed, which could provide potential diagnostic biomarkers and therapeutic interventions for type 2 diabetes (Ma et al., 2022).

#### 4.3.3 SCS and developmental biology

The emergence of SCS has greatly promoted research progress in the field of developmental biology. Firstly, SCS has been effectively utilized in the identification and classification of cell types at different stages of development. By determining gene expression patterns, specific cell subsets during development can be identified. Previous studies using SCS have demonstrated that cells with similar gene expression profiles can be grouped together and used to identify cells in multiple tissues and, more importantly, new types of cells within these populations (Zeisel et al., 2015; Zhou et al., 2016). Secondly, SCS can help delineate cell differentiation trajectories during the differentiation process. Specifically, trajectories of cell differentiation could be inferred and constructed by analyzing differences in gene expression in cells at distinct stages. An earlier study distinguished different human ES cell-derived progenitor states via scRNA-seq analysis of 1776 cells. Novel regulators of transition from mesoderm to endoderm were validated by reconstructing the different trajectories at single-cell resolution (Chu et al., 2016). In addition, application of SCS in reproductive development has been a hot topic in recent years. More recently, scRNA-seq analyses of the early developmental stages of mammalian and vertebrate embryos have been conducted. Researchers have used SCS to track the embryonic development of zebrafish and frogs, and construct dynamic maps of gene expression, thereby uncovering the entire process by which a single cell can generate an entire organism (Farrell et al., 2018; Han et al., 2018; Wagner et al., 2018).

Overall, the evolution of SCS provides powerful tools and methods in the field of developmental biology. Using SCS, we can gain insights into the cell types and differentiation trajectories during development, and identify the specific mechanisms underlying cell fate decisions. These advances will further advance the study of developmental biology, and expand our understanding of the origin and evolution of life.

#### 4.3.4 SCS and microbiology

Important advancements in microbiology have also been attributed to single-cell sequencing. SCS is widely used in this field to gain insights into microbial diversity, function, interactions, and evolution. Firstly, due to the low gene content and small sample numbers of microorganisms, conventional sequencing methods cannot be employed to sequence microorganisms that are difficult to culture. In comparison, SCS can be applied to sequence individual microbial cells with a high degree of accuracy, thereby revealing new microorganisms and further clarifying microbial life processes. For instance, DeLorenzo S et al. (DeLorenzo et al., 2012) sequenced a rare marine microbe, showed its association with sulfur oxidation, and further identified the genes involved in aerobic metabolism. Secondly, SCS could be applied to obtain information about metabolic phenotypes and genotypes of microbial single cells. In a previous study, single-cell microorganisms with specific metabolic phenotypes were isolated, and single-cell genome sequencing of this complex microbial system achieved. This method was used to simultaneously obtain phenotype and genotype information on the target microorganisms, and genome integrity reached up to 93% (Jing et al., 2021). In addition, SCS may be used to explore interactions and cooperative behaviors between host and organism. For example, earlier reports have revealed host-microbe interactions at spatial, cellular, and molecular levels in oral squamous cell carcinoma and colorectal cancer by applying *in situ* spatial analysis and single-cell RNA sequencing techniques (Nino et al., 2022). Finally, SCS technology effectively facilitates the identification of subtle variations in microbial genomes, study of evolution and tracing of the origin of pathogenic microorganisms. Further investigation of viral infection dynamics and redefinition of the metabolic profile, pathogenic potential, and drug resistance of pathogenic microorganisms may also enable the timely and accurate diagnosis of certain rare infectious diseases (Tolonen and Xavier, 2017). In summary, application of SCS provides new perspectives and methods for the study of microbiology, leading to key information on microbial diversity, interactions, and evolution, and consequently, significant research developments.

### 4.4 Advantages and limitations

This report has provided a comprehensive compilation of data from SCS studies between 2010 and 2022, including publication volume and growth patterns, journals, authors, institutional connections, references, and keywords. Furthermore, we simultaneously used three bibliometric approaches, including VOSviewer and CiteSpace, both widely recognized in the bibliometrics community, which improved the validity of our data analysis procedure.

This study inevitably has a number of limitations that should be taken into consideration. As the scope is limited to English language writing, representation of non-English publications may have been insufficient. Additionally, exclusive use of the WOSCC database may have resulted in the exclusion of essential studies from other databases. Moreover, insufficient data prevented the inclusion of all papers published in 2022.

## 5 Conclusion

Using WOSCC as a database, we employed CiteSpace software for bibliometric and visual analysis of global research on SCS spanning the previous 12 years, with the aim of providing a clear scientific summary of the development trends in this field. Visual analysis disclosed that studies on SCS are in a stage of rapid development and relevant literature is constantly emerging, showing a stable growth trend. The United States, China, Germany, England, and other nations have made significant contributions to the area of SCS research. The journals, organizations, and authors with the most influence included *Nature*, Harvard Medical School and Regev A, respectively. COVID-19, tumor microenvironment, scRNA-seq, and neuroscience factors were identified as hot topics in SCS research. Currently, a period of rapid development in SCS technology continues to drive the common progress of multiple disciplines. Evidently, problems with single-cell sequencing remain, such as amplification bias and difficulty in processing sequencing data. However, with continued advancements in SCS technology, we expect its application scope to increase and the depth and accuracy of analysis to further improve, with the ultimate goal of implementation in the diagnosis and treatment of multiple diseases.

## Data Availability

The original contributions presented in the study are included in the article/Supplementary Material, further inquiries can be directed to the corresponding authors.

## Abbreviations

SCS, Single-cell sequencing; scRNA-seq, single-cell RNA sequencing; WOSCC, Web of Science Core Collection; SCP, single country publications; MJP, multinational joint publications; JCR, Journal Citation Reports; IF, impact factor; SCLVM, single-cell latent variable model; HiPF, Hierarchical Poisson Factorization; SPLitseq, split-pool ligation-based transcriptome sequencing; TME, tumor microenvironment; aEC, activated endothelial cell.
