# Supplementary material for: Bibliometric and visual analysis of single-cell sequencing from 2010 to 2022
Source: Front Genet. 2024 Jan 11;14:1285599. doi: 10.3389/fgene.2023.1285599 (PMC10808606; doi:10.3389/fgene.2023.1285599)
Supplement: Supplementary file 1 [file DataSheet1.docx]

Supplementary Material

# Supplementary Data

All analysis codes have been uploaded in the form of attachments

# Supplementary Figures

**
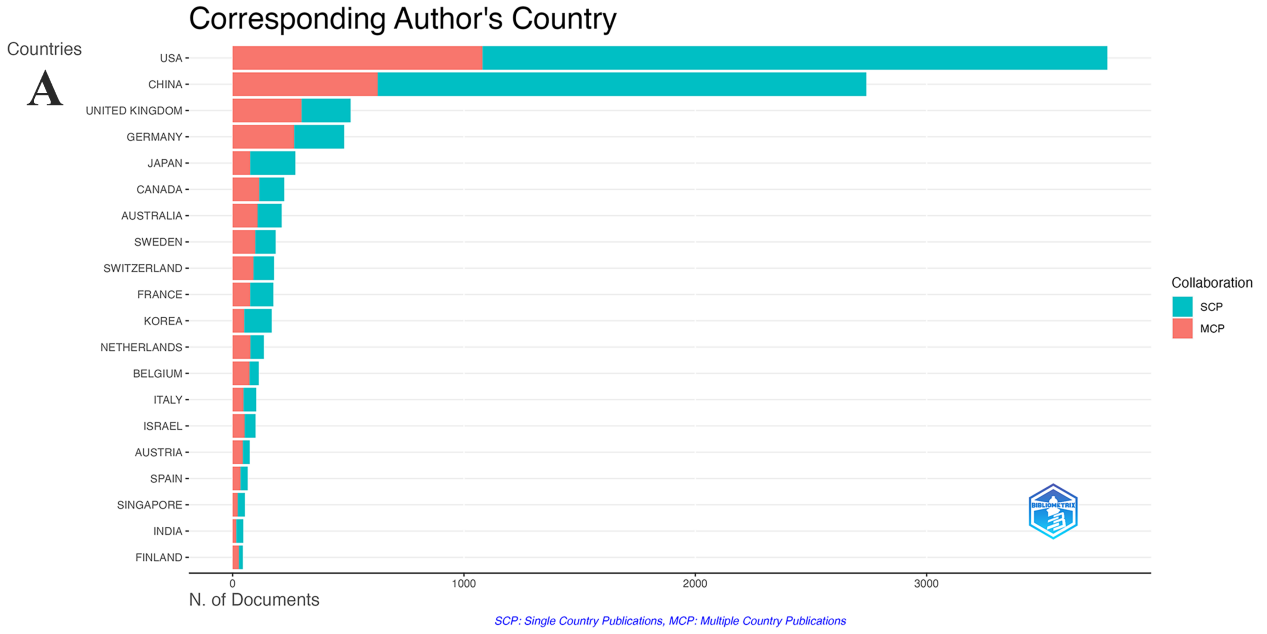
**

**
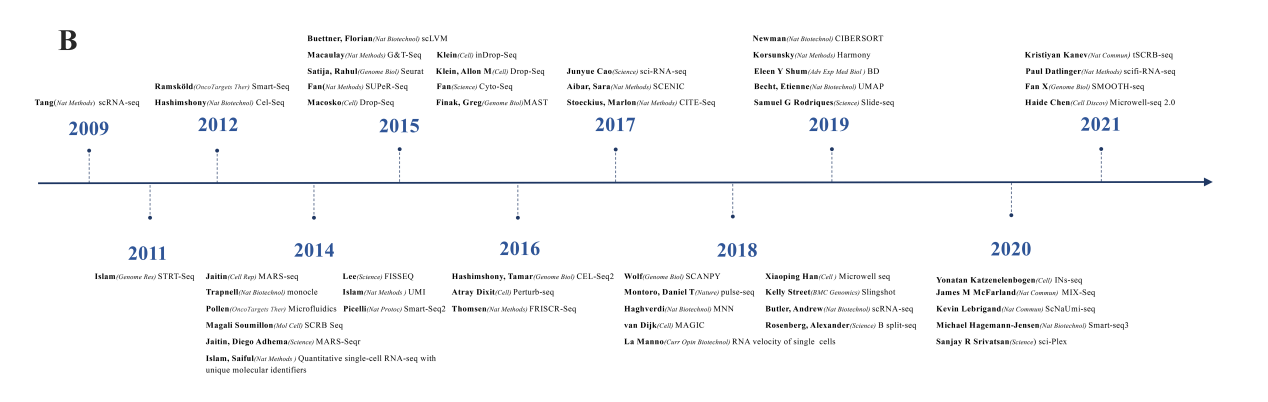
**

**Supplementary Figure S1. Analyses of single-cell relevant authors.** **(A)** Histogram of the top 20 countries distribution of SCS-related authors. **(B)** Time axis of SCS technology development history from 2009 to 2021.


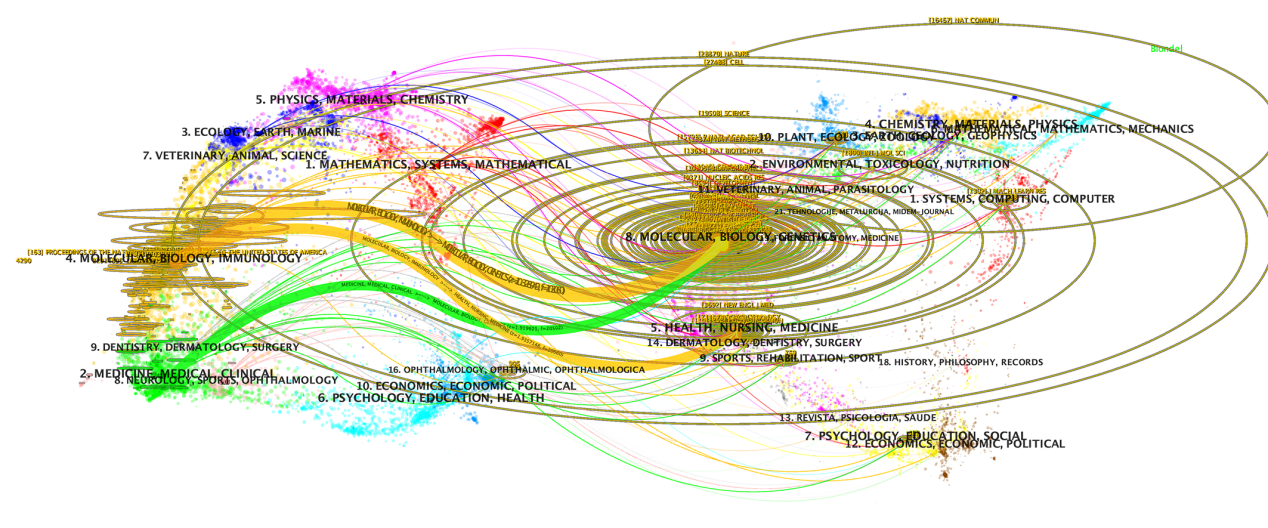


**Supplementary Figure S2 Analyses of single-cell relevant journals and co-cited academic journals.** The journal-overlay dual-map. Curves represent the citation line. The citing journals can be found on the left and the cited journals can be found on the right. The vertical axis becomes longer in proportion to the number of papers journal publishes. The elliptical horizontal axis gets longer as there are more authors.


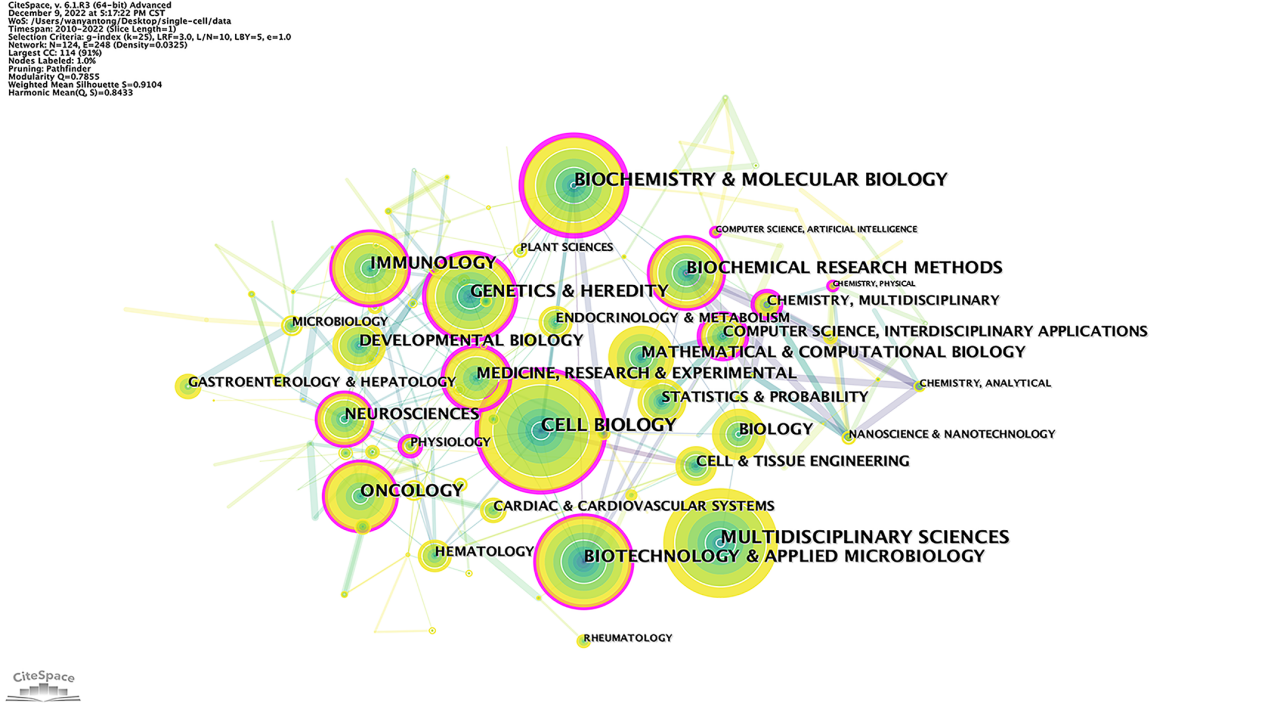


**Supplementary Figure S3 Analyses of SCS correlation subject.** The purple circle signifies a greater centrality and significance of the subject.
